# Education for Sustainable Development: Challenges for Postgraduate Programmes

**DOI:** 10.3390/ijerph20031759

**Published:** 2023-01-18

**Authors:** Ángel Acevedo-Duque, Carmen Jiménez-Bucarey, Tohtli Prado-Sabido, Mirtha Mercedes Fernández-Mantilla, Irene Merino-Flores, Sandra Sofía Izquierdo-Marín, Nicolás Valle-Palomino

**Affiliations:** 1 Programa Doctorado Ciencias Sociales, Universidad Autónoma de Chile, Santiago 7500912, Chile; 2 Vice-Rectorate of Quality Assurance, Universidad Andrés Bello, Santiago 7550000, Chile; 3 Faculty of Accounting and Administration, Autonomous University of Sinaloa, Culiacan 80020, Mexico; 4 Facultad de Ciencias de la Salud, Escuela de Psicología, Universidad César Vallejo, Trujillo 13001, Peru; 5 Escuela de Posgrado, Universidad César Vallejo, Trujillo 13001, Peru; 6 Programa de Estudio de Psicología, Facultad de Medicina Humana, Universidad Privada Antenor Orrego, Trujillo 13011, Peru

**Keywords:** SDG challenges, SDG targets 4.7, sustainable education, education for sustainability, higher education, sustainable training

## Abstract

As the world faces progressive and interconnected global crises and conflicts, the educational expectations set out in the 2030 Agenda for Sustainable Development are in jeopardy. With the COVID-19 pandemic in its third year, the war in Ukraine has exacerbated the food, energy, humanitarian, and refugee crises, all against the backdrop of an unfolding climate emergency. The aim of this research is to analyse the challenges faced by postgraduate programmes in training human talent for sustainable development on the basis of Grounded Theory. To do so, we have used a dialogical intervention through the complementary experiences of authorities of higher-education institutions that live day by day for a fair, quality, and sustainable education. With a naturalistic qualitative method, where the hermeneutic analysis procedure is structured in five phases, and with data from key informants from 9 countries, 20 interviews are obtained with key informants in Latin American and Spanish universities during 2021, according to inclusion criteria such as: belonging to a higher-education institution, with a doctorate degree, with more than 10 years of experience in management, and training in postgraduate programmes. The data are processed through ATLAS.ti9, which allows for the analysis of the key informants’ discourses. The findings show that the university institutions that currently offer postgraduate programmes are considering improving the quality of education; the first challenge is to redesign the curricula according to the demands of the current and future world, incorporating technological resources and knowledge of the environment; inter- and transdisciplinary curricula that form enterprising postgraduates with a solid ethical life project; critical, complex, and systemic thinking.

## 1. Introduction

Using current data, the 2022 Sustainable Development Goals Report provides evidence of the destructive effects of these crises on the achievement of the Sustainable Development Goals. Higher-education institutions (HEIs) have a key role in shaping societies towards a more sustainable future through a roadmap for the implementation of the 2030 agenda, especially Goal 4, which aims to ensure inclusive and equitable quality education and promote lifelong learning opportunities for all [1] (Pereira and Moura, 2019). Target 4.7 specifically addresses education for sustainability; however, creating a monitoring and evaluation framework for target 4.7 has been a challenge at this time.

Currently, educational challenges play a synergistic role in achieving the aspirations included in the 2030 Agenda and the Sustainable Development Goals (SDGs) [2], ensuring that all learners acquire the knowledge and skills needed to promote sustainable development (United Nations General Assembly, 2015: 17) [3], and calling for transformative educational change around the world.

In addition, target 4.7 offers many opportunities for the world, such as: giving legitimacy to development education (DE), global citizenship education (GCE), and education for sustainable development (ESD) [4,5,6], which are becoming significant challenges at the moment; connecting practitioners with a diverse global community of educators committed to social justice and sustainability; finally, creating a platform to showcase the impact of DE and related education.

Higher education (HE) must renew its pedagogical paradigm by placing students at the centre of the teaching–learning process [6]. In order to promote effective training programmes, many types of pedagogical innovations are emerging in higher education, some of which emphasise and provide experiential and participatory learning scenarios aligned with a strong ethical commitment, in the hope of making a significant contribution to sustainable development [7].

In addition, the curriculum and objectives of higher education have been reformed in most countries of the world in recent decades [8]. The aim of the new HE curricula is to develop actively educated individuals who possess the knowledge, skills, and confidence to effectively apply their learning, rather than just educating meaningless content [8]. In this regard, a number of constructivist-based pedagogical models have increasingly attracted the attention of researchers, teachers, and students, such as active methodologies based on social inclusion, service-based learning, healthy habits, and well-being.

However, based on the comments of the United Nations General Assembly (2015), and making comparison with the results issued in the 2022 Sustainable Development Goals Report on SDG 4 and target 4.7, the COVID-19 pandemic deepened the crisis in education, with severe disruptions in education systems around the world [5,6]. The closure of education institutions has had worrying consequences for children’s learning and well-being, especially for girls and the disadvantaged, such as children with disabilities, rural dwellers, and ethnic minorities. It is estimated that 147 million children lost more than half of their classroom instruction in the last two years. As a result, this generation could lose a combined total of USD 17 trillion in lifetime earnings (in present value).

Governments need to put in place ambitious programmes to ensure that all young people have a quality education with a vision for sustainable development, and that they make up for lost learning and can meet their psychosocial needs in order to move quickly into academic university and vocational training. The greater the absenteeism in these relevant subjects, the less likely they are to contribute to the future [5,6].

Students from more disadvantaged backgrounds are at greater risk due to socioeconomic factors, such as the need to generate income, increased caring responsibilities, and early and forced marriages [5]. Those who were unable to access distance learning during confinement are also at greater risk of dropping out of education [1,2,5,8]. This raises challenging questions: What are the “knowledge and skills necessary for sustainable living” and who decides which are most important? What kind of educational programmes are needed to develop the knowledge and skills necessary for sustainable living and global citizenship? How will we know if these programmes are working? [7,8].

Therefore, to make progress in this challenging area, we need to generate actions such as: (i) cooperation and exchange between the various “educations” mentioned in Goal 4.7; (ii) a critical dialogue between North and South on the complex nature of global citizenship; (iii) the recognition that progress towards Goal 4.7 requires diverse approaches to global citizenship; (iv) the recognition that progress towards Goal 4.7 requires diverse approaches to the development of sustainable livelihoods and global citizenship to measure results; (v) respectful conversations between practitioners, policy makers, and donors, especially on the use of targets as a means of learning and development, rather than as a means of control [9].

On the other hand, well-trained human capital can be seen as the engine of the productive process of new discoveries, ideas, development, and, ultimately, new value-added outputs [10]. In recent years, numerous studies have been carried out as a result of the widespread interest in education [10,11,12,13]. Moreover, the United Nations emphasised that education for all is always an inseparable part of the agendas of both the Millennium Development Goals (MDGs) and the Sustainable Development Goals (SDGs) [14].

In this broad sense of the epistemology of Goal 4 of the SDGs for this research, the vision of the main heads of higher-education institutions, research groups, and representatives of UNESCO in Latin America and Spain as the units of analysis is evident. For this reason, the research question is: What are the postgraduate challenges for Higher-Education Institutions in the training of human talent for sustainable development? Furthermore, they are defined as guarantees of quality, inclusive, and equitable education to promote learning opportunities and opportunities for all. It is for this reason that the purpose of this study is to show the challenges of postgraduate education in the training of human talent for sustainable development from the vision of representatives and authorities of postgraduate programmes from different houses of study.

As mentioned above, the issue of education for sustainability is one of the greatest challenges facing Latin America and Europe and whose most affected populations are mainly interest groups. For this reason, the paper is structured as follows: after the introduction, a review of the literature framing the study and the purpose of the paper are presented, explaining the grounded theory as a method to establish the importance of the theoretical constructs derived from the contexts and, therefore, from the problems or areas of interest limited to that context. The results are then discussed, demonstrating the relevance of sustainability education to sustainability education, and, finally, the discussion presents the conclusions of the study.

## 2. Background

### 2.1. Goal 4 Targets for the Development of Sustainable Quality Education in Postgraduate Programmes

The COVID-19 pandemic deepened the crisis in education, with severe disruption to education systems around the world [15]. The closure of higher-education institutions has had worrying consequences for the learning and well-being of postgraduate participants, especially for those who live far away from their place of study.

**Figure 1:**
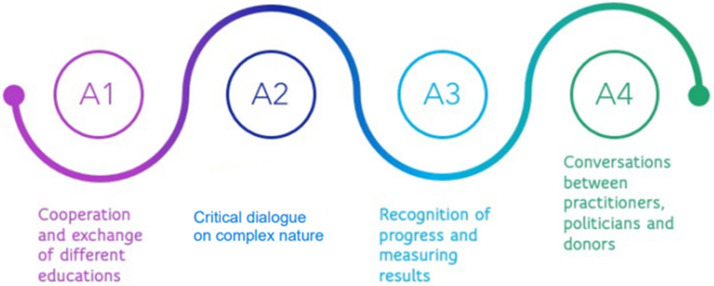
Action Generation Model According to the United Nations General Assembly.

In this worrying sense, world leaders signed up to the 2030 Agenda for Sustainable Development, a structured agenda with a focus on some of the goals, which is to achieve accessible education for all, the foundation for sustainable development and peace [15]. Through partnerships, policy guidance, capacity development, monitoring, and advocacy, UNESCO has coordinated the international community’s action towards the achievement of SDG 4 on education and has developed a roadmap to achieve the Education 2030 Framework for Action (see Figure 1) [16].

Faced with challenging contexts, higher-education institutions, especially in postgraduate programmes, must respond with resilience to reflect and develop planned and concerted strategies to maximise the current potential and take up the challenge to “innovate in processes that aim at sustainable social developments”, for which they must assess the academic and managerial effects that have been experienced during confinement, in order to define and adapt their management and research models and curricular designs in accordance with the goals of Goal 4 [17].

The realisation of quality education is the basis for improving people’s lives and sustainable development [2]. Significant progress has been made in improving access to education in postgraduate programmes aimed at promoting sustainable development, especially for women. However, according to the latest SDG report, it specifies that the COVID-19 pandemic deepened the crisis in education, with severe disruption to education systems around the world. The closure of educational institutions has had worrying consequences for learning and well-being [18].

Before COVID-19, the world was not on track to achieve these goals, and the pandemic reversed some of the gains made in education [6]. Around the world, education was severely disrupted, particularly affecting the most vulnerable learners. However, the crisis allowed for the strengthening or creation of global partnerships to rethink the future of education and achieve the Education 2030 goals [6,19,20].

While governments have the primary responsibility for ensuring quality education, the 2030 Agenda represents a universal and collective commitment. Within this framework, UNESCO is leading the secretariat of the Global Fund for Education Partnership and the Education Transformation Summit to be held in September 2022 [21,22,23,24]. These two examples are based on strengthening and coordinating the actions of different UN agencies and key partners to achieve SDG 4 and its corresponding targets. The fact that the Secretariat brings together many agencies ensures that each takes ownership of the work that has been done and is a powerful springboard for sustainable achievements towards achieving the 2030 Agenda.

### 2.2. Challenges in Postgraduate Education for Sustainable Development

Since the United Nations Conference on Environment and Development held in Rio de Janeiro, Brazil, in 1992, a framework was created for concerted action and the search for solutions to the environmental problems that afflict our planet at the global, regional, and local levels [25]. In it, government leaders, business leaders, and representatives of civil society generated basic working agreements that have been refined over the years and in which the ideas of “sustainable development” acted as their guiding nucleus, making it possible to achieve some goals over the years and posing new challenges that promote knowledge as a result of processes of searching for, organising, recovering, and communicating information [25].

From this derives the interaction and elaboration of social and cultural strategies necessary for this knowledge to be shared and used by people in different contexts and situations [26], especially those perspectives in the academic world of the 21st century, which require public, private, and social organisations to adopt innovative strategies in line with the challenging demands of the environment [26,27]. In view of the above, university organisations in their management of traditional postgraduate programmes give way to the incorporation of mechanisms that provide responses to the development of the world of education in a more sustained manner, with the aim of becoming intelligent, proactive, dynamic, creative, and decentralised organisations where the competences of human talent are the cornerstone for the achievement of the objectives that the institutional [28].

It is evident, then, that, through the management of their programmes, universities show an important interest in sustainable development and aspire to create strategies and instruments that allow the integration of natural heritage conservation and development to satisfy basic human needs, promoting equality and social justice, social self-determination, cultural diversity, and the preservation of the integrity of ecosystems [23]. In addition, the agreements of global academic organisations have demonstrated an increasingly clear understanding that the planet’s resource base is subject to a high rate of erosion that threatens the survival of a multiplicity of existing species, including the human species, as well as the functional stability of the planet.

## 3. Materials and Methods

**Figure 2:**
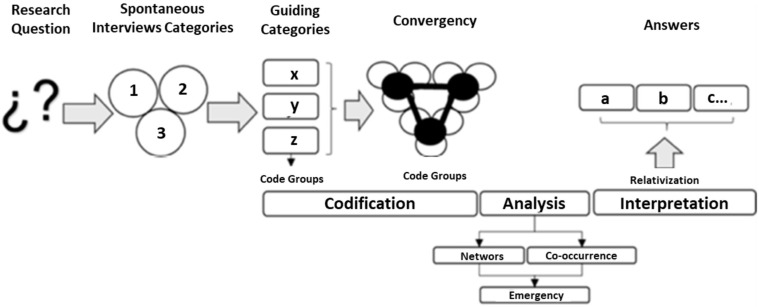
Hermeneutic analysis in 5 phases of González-Díaz et al. (2021) [31].

The qualitative research methodology recognises the human being as a producer of knowledge to understand reality through the construction of “meanings”, rescuing the heterogeneity of society [5,29]. Its methodological structure is neither linear nor previous; it emerges in the development of the study through an inductive process reconstructing the realities of the subjects investigated from their natural environment. For this study, the phenomenology and fundamental theory of Heidegger and Strauss trace an argumentative axis towards the defence of the specific character of human reality in the face of the challenges of sustainability that makes it irreducible to the categories of analysis of physical reality whose essence are objects or material things. Therefore, this qualitative research with a naturalistic approach was structured following the procedure of [30,31], who configured the hermeneutic analysis in 5 phases (see Figure 2).

For this purpose, we used a dialogical intervention through the complementary experiences of authorities and academics from higher-education institutions, who, to this day, manage framework agreements, international partnerships, teaching, and cooperation networks for research projects, who live day by day for a fair, quality, and sustainable education. With this post-positivist methodology, the hermeneutic analysis procedure is structured in five phases and with the information provided by key informants from 9 countries, obtaining 20 interviews carried out in Latin America and Spain during 2021, according to inclusion criteria such as: belonging to a higher-education institution, with a doctorate degree, with more than 10 years of experience in management, and training in postgraduate programmes. The data were processed through ATLAS.ti9, which allowed us to analyse the discourses of the key informants.

The research methodology refers simultaneously to the way in which problems are approached and answers to them are sought. In qualitative research, it is considered that reality can never be apprehended in its entirety and requires the existence of a cognitive subject influenced by its cultural aspects and individual social relations, which make epistemic reality dependent, for its definition, understanding, and analysis, on the knowledge of the ways of perceiving, thinking, feeling, and acting on these cognitive subjects [30,32].

Stage 1. Problem Question (PP): The study answers a single question that encompasses the problem and directs the course of the investigation. The methodology is applied according to the expectations of each informant. What are the challenges faced by postgraduate programs in the training of human talent for sustainable development?

Stage 2. Categorical Spontaneous Interviews (EEC): The construction of the EEC is based on two approaches that direct the research route for its analysis. These are applied to key informants to determine guide categories.

Stage 3. Guiding categories (CO): Take into account the responses of 20 key informants from different institutions both in Europe and Latin America according to the following criteria: more than 5 years of experience in academic teaching, doctoral teaching, experience in deanships and postgraduate studies, and research vice-chancellors; later, a word cloud is created to determine a coding by group and establish the guiding categories that respond to the challenges faced by postgraduate studies for sustainable development. The distribution of the unit of analysis is presented in Table 1.

Stage 4. Convergence (C): The convergence of informants is grouped according to the categories oriented by citations and open codes. With this, a semantic network is established where the rooting and density of each category are highlighted, in order to subsequently evaluate the co-occurrence index through group codings that are represented with a Sankey diagram. Finally, the calculation of the emergence index is established, which indicates the average range of mentions of rooting and density, in order to determine those indicators that are above the means and thus able to give an interpretation of the results.

Stage 5. Analysis and interpretation of results (AIR): Using the Atlas.Ti9 software, open-source semantic networks, rooting and density tables, Sankey diagrams, and emergence calculations are obtained to respond to the objective of the study (see Table 1).

## 4. Results

**Figure 3:**
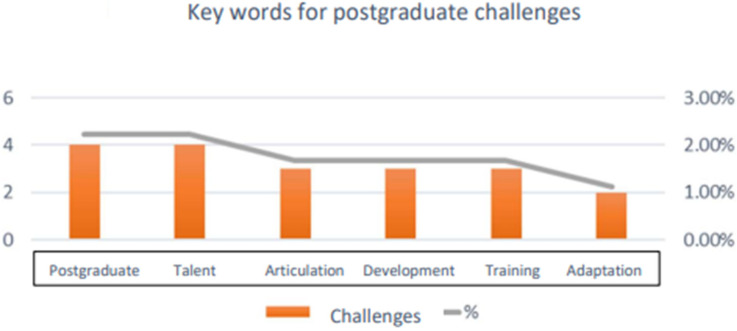
Word cloud results. Own source, 2022.

This section presents the results of the interpretative hermeneutic analysis of the interviews with key subjects from different universities in Latin America and Europe. The interviews in the units (such as deans, rectorates, and specialists in postgraduate programs) of the different university institutions were obtained according to inclusion criteria, such as: university affiliation, 10 years of research and postgraduate experience, more than 30 years of age, doctoral level of university studies, and experience in designing postgraduate programs (see Figure 3).

### 4.1. Guiding Categories

A category is a research topic that is taken into account to categorise qualitative information and validate it by analysing the results of the information. González-Díaz et al. (2021) [31] stated that categories are a kind of conceptual drawer in which information is stored. After deducing the theoretical references in order to deduce the guiding categories and previously organising the information, the guiding categories were extracted according to the discourse emitted by the key informants [32].

This section shows the categorisation process in this research, taking into account the qualitative information from the key informants, and then validating it by analysing the results of the information. González-Díaz et al. (2021) [31] stated that categories are a kind of conceptual drawer in which information is stored. After deducing the theoretical references to deduce the guiding categories and previously organising the information, the guiding categories were extracted according to the discourse emitted by the key informants [32].

Taking the answers of the key informants, an analysis was carried out to create the word cloud and its graphic representation, allowing for the determination of the guiding categories.

Table 2 shows the logical order obtained from the key informants and is used to apply the five-phase hermeneutic analysis method proposed by González-Díaz et al. (2021) [29]. This method contains three approaches (development and training of talent, research on sustainable programs, and resilience) that respond to the objective of the research; nine guiding categories, which govern the direction of the study; finally, nine groups of codes for the categories, which seek to detect the factors involved in this research. The last column links the contributing SDGs to the code groups.

### 4.2. Postgraduate Challenge Results

**Figure 4:**
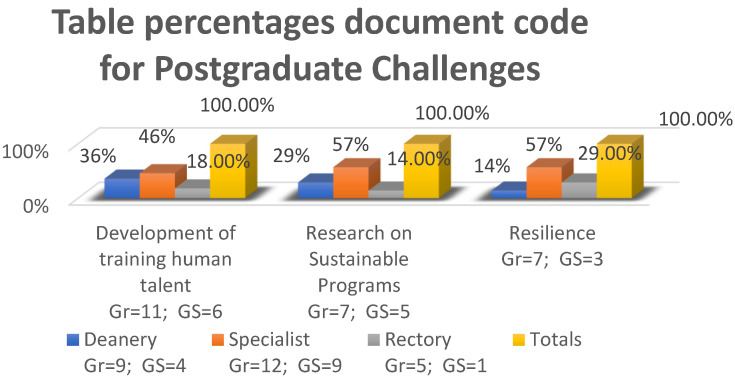
Percentage Code-Table of Documents for postgraduate challenges. Own elaboration, 2022.

Figure 4 details the three guiding categories that refer to postgraduate challenges, where the informants reflect the percentage of importance they give to each category, through the number of citations, such that for:

- Development and Training of Human Talent (DTH), 36% were obtained by the deans, 46% by specialists, and 18% by the rectory.
- Sustainable Research Program (SPR), 29% by deans, 57% by specialists, and 14% by rectorate.
- Resilience (R), 14% in deans, 57% in specialists, and 29% in rectorate (see Figure 4).

**Figure 5:**
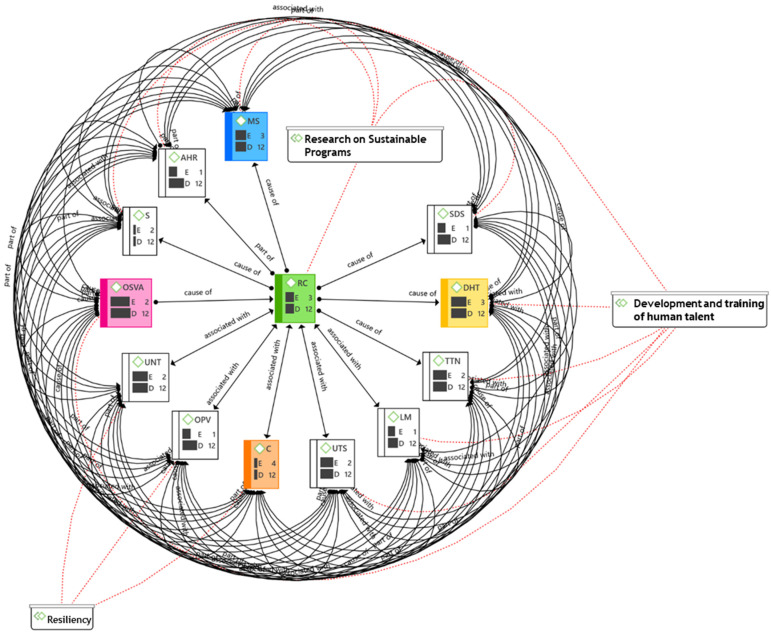
Semantic network of the postgraduate challenges category. Own elaboration, 2022.

Through the semantic network (Figure 5), the open codes are related to each other in a way that allows visualizing the perspectives of each informant in terms of both the guide categories and the groups of document codes. Thus, for the deans, specialists, and rectorates, the high expectations were focused on the Detection of Human Talent (DHT), Classroom Assistance (CA), and Virtual Class Assistance (VCA), as well as improvements in research programs (IP) (see Figure 5).

Speeches by rectors highlight the main challenges facing education for sustainable development, such as:

“To go hand in hand with the competitive world in order to be able to respond to the diverse needs”:

R-1.

“Generation of jobs from spin-offs, internationalization”:

R-2.

“Articulation of postgraduates with industry, although postgraduates can be academic and professional, we should always be able to generate a better articulation with the world of work and industry. articulation with the world of work and industry”:

R-3.

“Implement quality assurance systems that are in line with the times we live in the times we are living in”:

R-3.

“Gamify training, which translates into increased creativity and, in turn, productivity. productivity”:

R-4.

Speeches by specialists highlight the main challenges facing education for sustainable development, such as:

“I think one of the biggest challenges is the detection of human talent that identifies with the organisations identifies with the organisations”:

S-1.

“Flexibility of curricula: to be able to adapt to changing times and the uncertainty created by the pandemic”:

S-2.

“Maintaining a leadership focused on motivation, which helps you to build loyalty and retain human talent”:

S-3.

Speeches by deaneries highlight the main challenges facing education for sustainable development, such as:

“Humanistic research as a component of all postgraduate areas”:

D-1.

“Promoting social awareness in the youth of the future”:

D-2.

“Improvements in their research programs”:

D-3.

“How to eliminate administrative barriers to achieve effective internationalization and virtual mobility of students”:

D-4.

“Research staff committed to the development of human talent”:

D-5.

“The assessment of postgraduate careers not only from the perspective of the specialization to adapt to the demands of the new markets, but rather to aim at the energization of the professions with a character that transforms the market and society”:

D-6.

“Generate movements in the sustainable development of SMEs through the implementation of clean technologies, achieve education for work and personal well-being”:

D-7.

“Strengthening of professional and teaching skills based on a diagnosis of teacher training needs”:

D-8.

**Figure 6:**
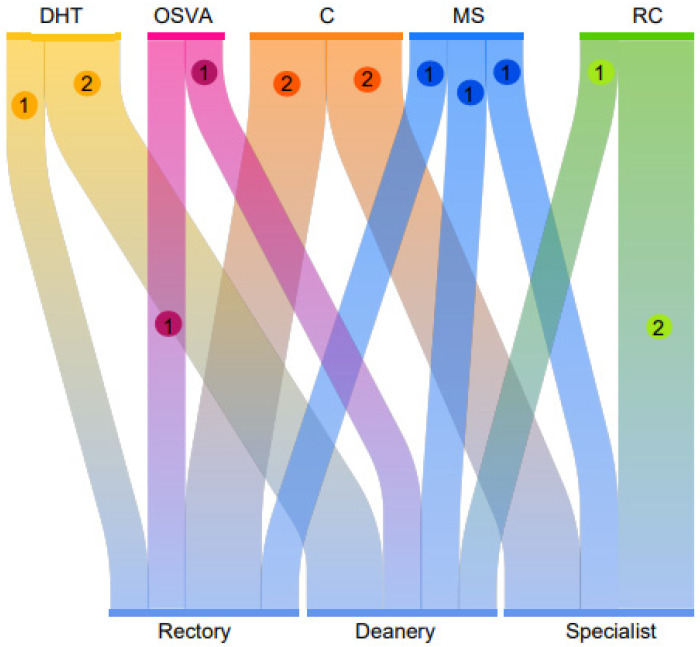
Sankey diagram. Own source, 2022. 1 = Absolute 2 = Relative.

The semantic network reflects all the codes involved for the analysis of postgraduate challenges where the emerging categories stand out for the highest density obtained, in such a way that competitiveness (C), the detection of human talent (DTH), multidisciplinary studies (MS), research commitments (RC), and face-to-face and virtual assistance (OSVA) are essential factors for consideration according to the opinions of the informants (see Figure 6).

Table 3 shows the percentage of participation of each informant with respect to the aforementioned categories:

- Research Commitments (RC) are supported by 66.67% by specialists and 33.33% by deans.
- Improvement of Research Programs (IPM) are supported by the same percentage with 33.33% for deans, specialists, and rectors.
- Onsite and Virtual Attendance (OSVA) is 50% for both deans and rectors.
- Detection of Human Talent (DTH) is 66.67% for deans and 33.33% for rectors (see Table 3).

**Table 3:** Shows the percentage of participation of each informant.

	Deanery Gr = 7; GS = 4		Specialist Gr = 9; GS = 9		Rectory Gr = 7; GS = 1		Totals	
Emergent Category	Absolute	Relative	Absolute	Relative	Absolute	Relative	Absolute	
● RC	1	33.33%	2	66.67%	0	0.00%	3	100.00%
Gr = 3								
● MS	1	33.33%	1	33.33%	1	33.33%	3	100.00%
Gr = 3								
● OSVA	1	50.00%	0	0.00%	1	50.00%	2	100.00%
Gr = 2								
● DHT	1	66.67%	0	0.00%	1	33.33%	3	100.00%
Gr = 3								

## 5. Discussion

Today more than ever, and as the COVID-19 crisis has shown, climate change and the environmental deterioration of the planet, people’s health, and social inequality are among the greatest challenges not only for higher-education institutions, but also for governments, businesses, and civil society. Higher-education institutions are creating learning strategies for students to acquire professional competences for a more sustainable future with a global vision, as part of the transition to the challenges of sustainability until 2030, where actions that contribute to the safeguarding of resources will be required [6].

The research question of the present project envisions the challenges facing postgraduate programmes in Higher-Education Institutions in the training of human talent for sustainable development, but this pandemic has undoubtedly forced them, especially, to reinvent themselves to generate new knowledge adapted to social, economic, and environmental needs [31,32]. Higher-education institutions have undergone changes in their management models and mission functions, allowing them to recognise that the objectives of sustainable development enable the significant achievement of quality education.

The results of the present research allow for recognising that through qualified key informants, the support of grounded theory will focus on emerging categories for an educational need for the future [31,32]. The findings point significantly to Research Commitment (RC), which is endorsed by key informants, 66.67% by specialists and 33.33% by deans. In Research Programme Improvement (RPI), there is an equal percentage of 33.33% for deans, specialists, and rectors, while face-to-face and virtual attendance (VPA) stands at 50% for both deans and rectors, as well as the detection of human talent (DTH) with 66.67% for deans and 33.33% for rectors. This demonstrates challenging scenarios in the implementation of a set of strategies, such as “the internationalisation of academic programmes to strengthen training and research in sustainable development” where teams and study networks are maintained with other institutions, offering postgraduate programmes, and governmental and non-governmental organisations, to carry out inter- and transdisciplinary collaborative work on problems prioritised in national and international contexts [33,34].

Collaborative work and research competences are key factors to adequately face the three challenges that Morín confers to knowledge to overcome its fragmentation: (a) a greater understanding of the global; (b) of the complex and its relationships; (c) of the uncontrolled and accelerated expansion of knowledge (Hernández and Cerinza, 2018), overcoming trivial research, and as Sime and Díaz (2019) stated, forming epistemologically educational thesis writers and researchers for sustainable development [33,35].

For Kopnina (2020) [19], the challenges facing university institutions for postgraduate programmes are to ensure inclusive and quality education by creating lifelong learning opportunities for all. This responds in some way to groups of codes such as: Research Commitments (RC), Research Programme Improvement (RIP), Face-to-Face and Virtual Assistance (VPA), and Detection of Human Talent (DTH). Codes that appear as a result of this research evidence and confirm the findings of authors such as Hota et al. (2019) [36,37,38,39].

The dynamic world of higher education and the challenges faced by postgraduate programmes are driving continuous improvement towards quality in postgraduate programmes for sustainable development, ensuring that the overcrowding of students does not contrast with quality [40,41]. In recent years, the increase in the number of masters and doctorates has not been associated with an increase in the impact research that society requires. Postgraduate studies should contribute to the training of professionals in research on sustainable programmes, and development and training of human talent and actions aimed at resilience, which can be given with different objectives, modes, and goals, in specialisation, masters, doctorate, and, more recently, post-doctoral programmes, taking into account the purposes of each [41,42,43].

## 6. Conclusions

The responsibility of higher-education institutions aims to improve quality not only in their management as houses dedicated to the training of professionals, but also to create an education that addresses the needs of today’s humanity, quality of life, wellbeing, and care for resources. The first challenge is to redesign curricula in accordance with the demands of the current and future world, incorporating technological resources and knowledge of the environment; inter and transdisciplinary curricula that form enterprising postgraduates with a solid ethical life project; critical, complex, and systemic thinking, who manage knowledge and metacognition and apply different research perspectives to contribute to improving the living conditions of the community. This process implies the permanent improvement of the teaching staff, and the continuous updating and evaluation of the professional training programmes, so that they are relevant to the social needs of production and sustainable development.

The above allows for the expansion of opportunities to focus research projects that contribute significantly to proposals that reduce the existing gaps in this context. This type of research not only allows us to show the current challenges in the training of human talent and its relevance for sustainable development, but also allows us to recognise that there are challenges and perspectives for this to be fulfilled under the requirements issued in the goals of the sustainable development objectives in the 2030 agenda that aim not only at educational quality, but also the care of well-being and resources.

### Limitations and Future Research

One of the limitations of this study is that it was conducted with a qualitative approach from grounded theory and only with the view of specified key informants—authorities, such as the research, management team, and teachers. The research project was carried out in the middle of the pandemic in 2021, interviewing key informants via the web, which perhaps prevented us from having more in-depth answers. However, the important work and support from authorities and specialists from higher-education institutions that aim at sustainability in a transversal manner, generating interest in having a significant impact on the economic, environmental, and social spheres, is noteworthy. Therefore, the opportunities aim to quantify the impact linked to the relationship with the student community, collaborators, the environment, and the different stakeholders in order to measure, manage, and enhance the impact that university cities have on this training need. Therefore, a qualitative study could help to identify the variables that could be most relevant for quality education focused on sustainable development. In addition, the study focused on showing the challenges that universities face in providing education focused on sustainable development. As such, future research could consider actual purchasing behaviour. Finally, it is important to be cautious in generalising the results, as the sample was mainly collected from key informants with a significant track record. Future studies could apply this method equally to the rest of the countries that make up Latin America and the European Economic Union and make an interesting comparison between countries or continents.

## Figures and Tables

**Table 1:** Distribution of unit of analysis.

Institution Represented	Country	Academic Degree	Position Held	Dependent Unit
Universidad Mayor de San Andres	Bolivia	Ph.D.	Director of Research and Graduate Studies	Rectory
Universidad Autónoma de Chile	Chile	Ph.D.	Vice President of Research and Graduate Studies	Rectory
Universidad Cattolica Santissima Concepción	Chile	Ph.D.	Director of Training Unit	Rectory
Universidad Nacional Andres Bello	Chile	Ph.D.	Postgraduate Coordinator	Rectory
Universidad Mayor	Chile	Ph.D.	Postgraduate Coordinator	Rectory
Universidad Católica de Colombia	Colombia	Ph.D.	Research Group Director	Specialist
Centro Int.de Investigación y Desarrollo	Colombia	Ph.D.	Director of Research and Development Center	Specialist
UNESCO	Colombia	Ph.D.	Advisor UNESCO-Colombia.	Specialist
Universidad de la Guajira	Colombia	Ph.D.	Doctoral Programs Professor	Deanery
Universidad Castilla la Mancha	Spain	Ph.D.	Professor of Doctoral Programs	Deanery
Universidad de Granada	Spain	Ph.D.	Professor of Doctoral Programs	Deanery
Universidad Autónoma de Madrid	Spain	Ph.D.	Professor of Doctoral Programs	Deanery
Universidad Nacional Autónoma de Honduras	Honduras	Ph.D.	Doctoral Program Director	Deanery
Tecnológico de Monterrey	Mexico	Ph.D.	Dean of Faculty	Deanery
Universidad Autónoma de Sinaloa	Mexico	Ph.D.	Research and Graduate Program Director	Deanery
Universidad Nacional Jorge Basadre Grohman	Peru	Ph.D.	Dean of Faculty	Deanery
Universidad César Vallejo		Ph.D.	Dean of Faculty	Deanery
Universidad Euroamericana	Panama	Ph.D.	Vice President for Research and Graduate Studies	Deanery
Universidad Dr Rafel Belloso Chacín	Venezuela	Ph.D.	Dean of Research and Graduate Studies	Deanery

**Table 2:** Guide to guiding categories, group codes, and their contribution to the SDGs.

Hermeneutic Approach	Category Guiding	Code Group	Contribution SDG	Specific Research
Challenges in postgraduate programmes	Human talent development and training	Teacher upgrading Face to face and virtual assistance Human talent identification Specialisation Leadership and motivation Teacher training needs	SDG 8 SDG 4 SDG 8	[19] [31] [19]
	Research and sustainable programmes	Research commitments Sustainable development of SMEs Applied and humanistic research Improvement of research programmes	ODS 8 ODS 4	[19] [19] [31]
	Resilience	Organisational policies and values Competitiveness Use of new technologies		

SDG 4 = Quality education. SDG 8 = Decent work and economic growth.

## Data Availability

Data are available on request from the authors.
